# Intervention to Prevent Child Custody Loss in Mothers with Schizophrenia

**DOI:** 10.1155/2012/796763

**Published:** 2011-11-01

**Authors:** Mary V. Seeman

**Affiliations:** Department of Psychiatry, Institute of Medical Science, University of Toronto, One King's College Circle, 7213 Medical Sciences Building, Toronto, ON, Canada M5S 1A8

## Abstract

Depending on jurisdiction, time period studied, and specifics of the population, approximately 50 percent of mothers who suffer from schizophrenia lose custody of their children. The aim of this paper is to recommend interventions aimed at preventing unnecessary custody loss. This paper reviews the social work, nursing, psychology, psychiatry, and law literature on mental illness and custody loss, 2000–2011. Recommendations to mothers are to (a) ensure family health (b) prevent psychotic relapse, (c) prepare in advance for crisis, (d) document daily parenting activities, (e) take advantage of available parenting resources, and f) become knowledgeable about legal issues that pertain to mental health and custody. From a policy perspective, child protection and adult mental health agencies need to dissolve administrative barriers and collaborate. Access to appropriate services will help mothers with schizophrenia to care appropriately for their children and allow these children to grow and develop within their family and community.

## 1. Intervention to Prevent Child Custody Loss in Mothers with Schizophrenia

A psychotic illness can, but does not need to, interfere with an individual's ability to be a good parent. Given well-timed, appropriate, and adequate education and resources, many individuals with psychotic illness succeed in parenting their children. This is not always recognized by child welfare workers who may continue to be influenced by outdated views of psychotic illness as intractable and parenting with schizophrenia as impossible [1]. Without effective intervention, parents who suffer from psychotic illness too often lose custody of their children [2], an unfortunate outcome that can be avoided by early intervention [3–5]. The emphasis in this clinical review is on *mothers* with a diagnosis of schizophrenia (because there is very little literature on fathers and effects on children merit a separate paper).

## 2. Method

This paper used the following grouped search terms in Google Scholar (which includes MEDLINE, EMBASE, PsycINFO, and SOCINDEX, as well as the nursing and legal literature) for the years 2000–2011: schizophrenia/diagnosis/custody; schizophrenia/impact/custody; schizophrenia/postpartum/custody; schizophrenia/termination parental rights. Following the literature review and case illustrations (from which identifying facts have been removed), recommendations are made for mothers, care providers, and policy makers.

## 3. Prevalence of Custody Loss in Mothers with Psychosis

Studying reports published in the last ten years, it appears that approximately 50% of women with schizophrenia who are mothers lose custody of their children, either temporarily or permanently, although the percentage varies by jurisdiction. For instance, a report from Canada indicates that 84% of parents treated for schizophrenia by a community treatment team were not living with their children at the time of interview [6], but this figure includes both male and female parents, so is probably higher than it would be for women alone. In London, UK, Howard and colleagues [7] established that 63% of women with psychosis (but only 26% of men) were parents. Hollingsworth [8] studied 322 women with serious mental illness and found that 26% had lost custody at some point in the child's life. A survey of mothers in psychiatric rehabilitative services [9] reported that 68% had been permanently separated from at least one child under the age of 18, often with little subsequent contact. The number of women with schizophrenia who experience custodial loss of their children is probably diminishing with time, as stigma lessens and interventions improve. Nevertheless, it remains high and effective intervention at the earliest stage of psychosis is warranted.

## 4. The Impact of Diagnosis on Custody Loss

Mothers with serious mental illness, as a group, fear that schizophrenia is equated in the mind of the public with parental incompetence or, worse, with parental neglect or violence [10]. This may well be the case because mothers with schizophrenia are often given relatively little opportunity to prove their parenting competence. Ackerson [11] has commented that parents with a diagnosis of psychosis are victimized twice first, by psychotic illness, then by protective removal of their children.

## 5. The Impact of Custody Loss on Mothers

Removing a child from a mother's care causes grief and distress to both. It is especially difficult for the mother when she has had little say in the process or when the event occurs at a time when she is too ill to understand what is happening. Diaz-Caneja and Johnson [12], in their qualitative study of 22 women with schizophrenia who were mothers, conclude that fear of losing child custody or access is always uppermost in the minds of severely mentally ill women, making it problematic to disclose to their care providers parenting difficulties that they may experience. Sands and colleagues [13] have reported that mothers with mental illness whose children are apprehended by child protection agencies are usually bewildered by events and confused about what steps to take in order to regain custody. Without psychiatric and legal guidance, they find it difficult to maintain contact with their children.

## 6. Postpartum Vulnerability to Custody Loss

The postpartum period is a particularly vulnerable time for women at risk of losing custody. Psychotic symptoms may emerge for the first time during this period and, for mothers with a prior history of mental illness, the risk of relapse is high during this reproductive phase [14, 15], bringing with it a very real threat of protective removal of children from the mother's care. Newborns are the most vulnerable members of society, and child protection legislation is therefore biased, as it needs to be, towards their needs rather than to the needs of the mother, however vulnerable she may also be. In order to meet the demands of competent infant care and retain custody of their infants, young mothers with severe postpartum psychiatric illness require substantial support and advocacy [16].

## 7. Composite Case Example of Unnecessary Children's Aid Involvement in Postpartum Psychosis

Patient A. was admitted to hospital for a postpartum psychotic episode. She had never been ill before. While she was in hospital, her infant was looked after at home by her parents. The baby's father was also involved in the child's care. Before the patient's hospital discharge, the psychiatric resident contacted Children's Aid, as a preventive measure, in order to notify the agency that Ms. A. might need help with mothering. Ms. A. had by then recovered and was functioning well. Psychiatric followup had been arranged, in addition to which several adults at home expressed willingness and availability to help look after the baby. The decision to call Children's Aid was solely determined by the fact that the patient had suffered a psychosis, placing her “on the books” of Children's Aid. While this could be perceived as a safety precaution for the family, it could also become a problem for the mother should, for example, the baby's father decide in the future to sue for sole custody of the child.

## 8. Causes of Termination of Parental Rights

The central moral and legal issue involved in temporary or permanent cessation of parental rights is the child's safety. When an environment is unsafe, the child must be removed until the situation changes [17, 18]. For small children, the safety of the environment is generally judged on the presence/absence of abuse and neglect. The parent must be able to provide basic care (shelter, nutrition, hygiene, clothing, and medical care) and security (protection from dangers, including unsafe people). As the child grows older, other domains of the parental environment take precedence. Brockington et al. [18] categorize these as the parent's ability to provide: *emotional warmth* (comfort, praise, and affection),* encouragement of learning* (through play, language, support of schooling, and social opportunities), *guidance and setting consistent limits (teaching consideration of others, self-discipline, *and* internal moral values), *and *a stable family base *for engagement with the wider world.

Assessing competent parenting requires skill and experience. While it is relatively easy to ascertain the presence of gross neglect or abuse, the more subtle qualities of parenting are harder to evaluate. Parent competency instruments are imperfect; they tend to focus on deficits rather than on strengths, and they are subject to cultural biases, since parental norms differ among cultures [19, 20].

## 9. Overrepresentation of Psychiatric Patients in Parental Termination Hearings

Besides parental competence, conditions such as physical and mental disability, side effects of medications, hospitalization history, quality and permanence of living arrangements, employment record, and socioeconomic status enter into decision making about custody. These variables are all intimately associated with mental illness, and, as a result, parents with psychiatric diagnoses are overrepresented in parental termination proceedings. In an Australian study, parental psychiatric illness was the most prevalent condition at such court hearings [21]. Marital status is also important—unmarried women (and this describes the majority of women with a diagnosis of schizophrenia) are more likely to lose custody than their married peers [22]. Social integration in a network of family, friends, and community members, often deficient in women with schizophrenia, is also relevant [8]. The more dense a social network is, the less likely it is for children to be apprehended by child protection services. The diagnosis of schizophrenia in itself undermines a woman's chances of retaining custody, so does the substance abuse that frequently accompanies mental illness [7]. Recent studies have shown that substance abuse is perhaps the most important contributory factor [23, 24], although there are many interacting factors that determine out-of-home placements. Young mothers suffering from psychosis find it very hard to disentangle themselves and their children from the web of problems in which they become caught.

## 10. Preventing Custody Loss: Recommendations for Mothers and Care Providers

There are several ways in which mothers with severe mental illness can reduce the risk of child apprehension. It is the responsibility of care providers to provide mothers with this information and training in order to help them to preserve the integrity of their family [25]. 

### 10.1. Maintaining Mental Health

In trying to provide for their children, mothers often neglect their own health and yet maintenance of personal health is a crucial first step toward ensuring child custody. This includes proper diet, a healthy sleep schedule, an exercise program, regular physician and mental health visits, and adherence to a prescribed regimen of medication. When questioned, most mothers with schizophrenia do understand that custody can be lost if prescribed treatment for their condition is not adhered to [26]. 

When women deny psychiatric illness, the intense desire to retain custody of their children can be used as a form of leverage, a controversial but effective strategy [26–28]. 

## 11. Case Example

Patient B., the sole caregiver of a 5-year old son, sought treatment for psychotic symptoms but refused antipsychotic medication. She was hospitalized against her will after she was verbally abusive to another mother at her son's school. During her hospitalization, her son was placed in the care of a cousin. In hospital, Ms. B. continued to refuse medication. She asked for a lawyer to represent her so that she could leave hospital and return to her son. The lawyer advocated for her with hospital staff and persuaded Ms. B. to agree to community treatment orders (outpatient commitment), which included monthly depot antipsychotic injections. The lawyer convinced her that this would be her best recourse in order to regain custody of her son. Ms. B. had an excellent therapeutic response to the antipsychotic and soon went home. Her son returned to her care, and, when the 6-month community treatment order expired, Ms. B. continued the injections voluntarily because she felt so much better. Child protection worked collaboratively with legal and mental health agencies to help this family stay together. Followup after many years showed that Ms. B. has succeeded as a parent. She has had no further hospitalizations, and her son remains in good health. 

As symptoms of psychosis decline, parenting stress is reduced and the quality of parenting inevitably improves [29]. Addressing symptoms alone is never sufficient [30], but does show the court that mothers are taking responsibility for this aspect of their recovery.

### 11.1. Self-Monitoring for Signs of Relapse

Avoiding recurrence of symptoms and the possibility of hospitalization is important for continuity of parental care, which means that psychiatric crises need to be avoided through anticipatory planning and self-monitoring. Mothers can be advised to maintain a written list of personal relapse triggers and early warning signs (sleeplessness, lapsed hygiene, increased suspiciousness, and so on) and to be knowledgeable about their medications. A requirement for dose changes whenever relapse threatens should be thoroughly discussed between mothers and care providers; the mother needs to know when and how she can increase (or decrease) her prescription to prevent a crisis. She needs to document what has worked in previous predicaments of a similar nature and what she can do to avert them. She needs ready access to crisis help. Evidence of self-monitoring convinces the court that mothers recognize that they suffer from a potentially relapsing illness and are doing their best to prevent recurrence.

### 11.2. Developing a Crisis Plan

Should hospitalization become necessary, it is important for mothers to be prepared for this disruption in their ability to care for their children. Several crisis plan templates are available electronically for parents with mental illness, none of which have as yet been evaluated for effectiveness [31]. It is best for all family members and all care providers to be involved in developing the crisis plan. The aim is to negotiate what needs to occur in an emergency and to clarify the responsibility of each member of the support network. Older children need to be part of the response team as they may be the first to notice their mother's early illness symptoms and they need to know whom to turn to under such circumstances. It is important, however, not to overburden children with the responsibility of looking after an ill parent.

The phone numbers and addresses of surrogate caregivers must be made available to children and also to care providers. Thought should be given to establishing backup caregivers in case of the unavailability of first choices. The plan should be written down, shared, and periodically updated because names and details will change. It should include critical information about the children's needs: their doctor, dentist, teachers, allergies, food and activity preferences, favorite toys, bedtime routines, and physical and psychological history. Plans for family pets should be included. Reupert and colleagues [31] report that it typically takes 6–12 months to develop a comprehensive crisis plan because all the necessary interagency meetings take that long to organize. Such a plan indicates to the court that mothers place their parental responsibilities above all else.

### 11.3. Taking Advantage of Parenting Resources

Depending on the community, parenting skills classes, parenting mutual aid or support groups, parent coaches, parenting warm lines, home visiting, and respite services may all be available resources [32–34]. An online parenting course has even been developed in The Netherlands [35]. Well-trained care providers should be able to point mothers in the right directions to access the best resources [36]. Upgrading parental skills demonstrates to the court that mothers are trying their best to be responsible parents.

### 11.4. Documenting Household and Child Care Routines

When asked by a judge to give evidence of good parenting, many mothers do not know what to say, especially because such questions are not usually asked of mothers unless they suffer from mental illness. The judge, however, is entitled to ask about safety issues and about issues pertaining to domains of parenting competence similar to those outlined by Brockington et al. [18] Mothers can be helped to document the day to day manner in which they address their child's instrumental and emotional problems, how they help their children resolve conflicts, how they set limits, and how they help to socialize their children. They need to build a record detailing their parenting strengths and the quality of the bonds that exist with their children. Care providers should be able to provide guidance for the mother so that she is not at a loss when questions about her parenting emerge in court.

### 11.5. Navigating the Legal System

Mothers need to understand the mandated child abuse reporting laws of their jurisdictions. They need to connect with attorneys who understand mental illness and family law and the family court system and who can act as strong advocates. Policies intended to promote a speedy resolution for children in out-of-home care may unintentionally discriminate against parents with mental illness because they fast-track the termination of parental rights, allowing only a brief time period for new parents to meet the goals set by child protection agencies. Attorneys and care providers need to help mothers achieve these goals as quickly as possible by ensuring access and legal rights to the necessary supports and services. Collaboration is important between child protection and lawyers who represent parents in custody and termination proceedings. It is not an easy collaboration, however, because child welfare professionals and court professionals come from two distinct cultures, the first a culture of care and concern, the second an adversarial system that, above all else, values individual rights [37]. There is a definite need for mental health education of judges and court professionals.

## 12. Recommendations for Policy Makers

Early intervention services, adult mental health services, and child protection services often act in competition rather than in cooperation [38]. It is crucial to develop a philosophy and care system that cooperatively addresses the needs of the whole family. There is now a promising evidence base of effectiveness of wraparound services for families impacted by serious mental illness [39]. 

The term, “wraparound,” is increasingly being used to describe a family-driven, strengths-based approach that uses an array of both formal services and natural supports [40]. Another phrase often used is “system of care.” A system of care is a network of structures and relationships that is held together by shared values and that operates across administrative and funding jurisdictions [41, 42]. A family-driven system of care is based on the needs of children, parents, and extended family. It supports choice, ongoing evaluation, and accountability and promotes partnerships between families and professionals, collaboration between multiple agencies and service sectors, and individualized services that are sensitive to cultural differences. The cultural sensitivity of a service refers to the ability of its staff to understand, value, and incorporate the perspective of the family into service provision and, whenever possible, provide services in the family's language of choice.

In a wraparound system, there is a single point of entry for the many services that are provided. Among these are early identification and prevention strategies, attention to reproductive and child health, substance abuse counseling, case management, liaison with schools and the legal system, financial support, crisis management, housing, transportation aid, vocational help, spiritual, cultural, and recreational guidance, and respite care. Ideally, the services are open ended and sensitive to the stigma associated with mental illness. Cook and Steigman [43] advocate supports specifically designed to preserve the parental relationship. They identify assessment of parenting strengths and needs, birth control counseling, pregnancy decision-making support, trauma and abuse counseling, peer support, parent mentoring, self-help, support groups for children, and medication management as important aspects of a system of care for families with a mentally ill member. Specific counseling around benefits and entitlements is also critical for low-income mothers, some of whom may be intermittently homeless and require housing support [44]. Administrative policies, training opportunities for service providers, and hard work on the part of mothers themselves are all needed to ensure that children of mentally ill parents grow up, whenever possible, in their family of origin.
